# Wettability of a Polymethylmethacrylate Surface by Extended Anionic Surfactants: Effect of Branched Chains

**DOI:** 10.3390/molecules26040863

**Published:** 2021-02-06

**Authors:** Qin Jiang, Yuechun Du, Lei Zhang, Wangjing Ma, Feng Yan, Lu Zhang, Sui Zhao

**Affiliations:** 1 Key Laboratory of Photochemical Conversion and Optoelectronic Materials, Technical Institute of Physics and Chemistry, Chinese Academy of Sciences, Beijing 100190, China; jiangqin16@mails.ucas.ac.cn (Q.J.); zl2558@163.com (L.Z.); wjma@mail.ipc.ac.cn (W.M.); 2 University of Chinese Academy of Sciences, Beijing 100049, China; 3 School of Chemistry and Chemical Engineering, Tiangong University, Tianjin 300387, China; 18702230201@163.com (Y.D.); yanfeng@tjpu.edu.cn (F.Y.)

**Keywords:** extended anionic surfactant, branched chain, polymethylmethacrylate, contact angle, adsorption

## Abstract

The adsorption behaviors of extended anionic surfactants linear sodium dodecyl(polyoxyisopropene)_4_ sulfate (L-C_12_PO_4_S), branched sodium dodecyl(polyoxyisopropene)_4_ sulfate (G-C_12_PO_4_S), and branched sodium hexadecyl(polyoxyisopropene)_4_ sulfate (G-C_16_PO_4_S) on polymethylmethacrylate (PMMA) surface have been studied. The effect of branched alkyl chain on the wettability of the PMMA surface has been explored. To obtain the adsorption parameters such as the adhesional tension and PMMA-solution interfacial tension, the surface tension and contact angles were measured. The experimental results demonstrate that the special properties of polyoxypropene (PO) groups improve the polar interactions and allow the extended surfactant molecules to gradually adsorb on the PMMA surface by polar heads. Therefore, the hydrophobic chains will point to water and the solid surface is modified to be hydrophobic. Besides, the adsorption amounts of the three extended anionic surfactants at the PMMA–liquid interface are all about 1/3 of those at the air–liquid interface before the critical micelle concentration (CMC). However, these extended surfactants will transform their original adsorption behavior after CMC. The surfactant molecules will interact with the PMMA surface with the hydrophilic heads towards water and are prone to form aggregations at the PMMA–liquid interface. Therefore, the PMMA surface will be more hydrophilic after CMC. In the three surfactants, the branched G-C_16_PO_4_S with two long alkyl chains exhibits the strongest hydrophobic modification capacity. The linear L-C_12_PO_4_S is more likely to densely adsorb at the PMMA–liquid interface than the branched surfactants, thus L-C_12_PO_4_S possesses the strongest hydrophilic modification ability and shows smaller contact angles on PMMA surface at high concentrations.

## 1. Introduction

Wetting phenomena on solid surfaces [1,2,3] have attracted considerable attention in the fields of adhesion, [4,5] oil exploitation, [6] flotation, [7] membrane distillation, [8] washing [9] and lubrication [10]. By adding surfactants, the wettability of solid surfaces can be regulated [11,12]. The surfactant molecules can y adsorb onto the air–liquid interface and solid–liquid interface, thus changing the surface tension and the interfacial tension of solid–liquid, respectively [13,14]. Consequently, the contact angle is determined by the surface tension and the solid–liquid interfacial tension varies.

It is well known that polymethylmethacrylate (PMMA) is one of the most widely used acrylate plastics because of its excellent aging resistance and biocompatibility [15,16]. PMMA is a weakly polar polymer compound that contains -CH_3_, -CO, and -OCH_3_ groups [17]. Therefore, surfactants can interact with PMMA surfaces through a variety of adsorption methods. Numerous conventional surfactants’ adsorption behaviors on the PMMA surface have been investigated and reported in the literature. Harkot et al. explored the adsorption behaviors of anionic surfactant (AOT) [18] and cationic surfactants (C_12_(EDMAB), BDDAB) [19] on the PMMA surface. They found that the wettability of PMMA surfaces by the surfactants depends on the surfactants’ concentration to a large extent. When these surfactants’ concentration is around CMC, the contact angle and PMMA–solution interfacial tension reach the minimum value. As the surfactants’ concentration continues to increase, the contact angle and interfacial tension change little. Zdziennicka et al. investigated the wettability of cationic surfactants (CTAB, CPyB), [20] nonionic surfactants (TX-100, TX-114) [21,22] and anionic surfactants (SDDS, SHS) [23] on the PMMA surface. It is found that the contact angles and the interfacial free energy of PMMA–liquid (*γ*_SL_) of these conventional surfactants gradually decreased with the increase in surfactant concentration, which indicates that the surfactants will adsorb on PMMA surface by hydrophobic interactions. In recent years, research has been published about the adsorption behaviors of zwitterionic surfactants [24] and cationic gemini surfactants [25] on the PMMA surface. The group of Zhang discovered some surfactants with specific wetting properties because of the introduced polyoxyethylene (EO) units or branched hydrophobic chains in their structures [26,27,28]. These special surfactants will interact with the PMMA surface via polar groups at low concentration, resulting in a slightly hydrophobic PMMA surface. However, these surfactants will transform their original adsorption behavior to hydrophobic interactions after CMC, therefore *γ*_SL_ decreases with the increase in surfactant concentration.

The extended surfactant is a kind of novel surfactant, which contains a polyoxypropene (PO) chain inserted between the hydrophobic alkyl group and hydrophilic polar group [29]. Chen et al. [30,31] found that the extended surfactants’ PO spacer results in an obvious rugby shape for the surfactant molecules at the air–liquid interface. This allows the extended surfactants to exhibit excellent interfacial properties for household cleaning. Owing to the polar and non-polar groups of extended surfactants, they may adsorb onto the PMMA surface through both polar interactions and hydrophobic interactions, resulting in a variety of modification behaviors on the PMMA surface by special structural extended surfactants.

However, there are few reports involving the wettability of extended anionic surfactants on the PMMA surface. In particular, the effect of branched extended anionic surfactants on the wetting of the PMMA surface needs to be further explored. Wu et al. [28] explored the adsorption of branched betaine and cationic surfactants on the PMMA surface. It was reported that the branched C_16_GPB, C_16_GPC, and C_16_G(EO)_3_PC have a stronger hydrophilization capacity than similar surfactants with a linear structure. However, the branched structure inevitably increases the difficulty of forming aggregations; therefore, the adsorption amounts of branched C_16_G(EO)_3_PB on the PMMA surface are lower than the linear C_16_(EO)_3_PB. Zhang et al. [24] utilized two branched betaines (BCB, BSB) to detect their wettability properties on the PMMA surface. In their study, branched benzyl at hydrophobic chain of BCB improves the surfactants’ molecular size, resulting in an increase in the turning concentration for the adsorption behavior. Gao et al. [25] investigated the adsorption behavior of branched cationic gemini surfactants (C3, C6) on the surface of quartz. The branched cationic gemini surfactants with enhanced steric hindrance will destroy the tight arrangement of the surfactant molecules at quartz. Therefore, gemini surfactant molecules could not form a bi-layer adsorption on a quartz surface after CMC.

Herein, different branched extended anionic surfactants (L-C_12_PO_4_S, G-C_12_PO_4_S, and G-C_16_PO_4_S) were utilized to evaluate their adsorption properties at air–liquid and solid–liquid interfaces by measuring their surface tension and contact angles, respectively. It is helpful to understand the influence of the surfactants’ branch degree on the wettability of a medium energetic polymer surface.

## 2. Experimental

### 2.1. Materials

**Scheme 1:**
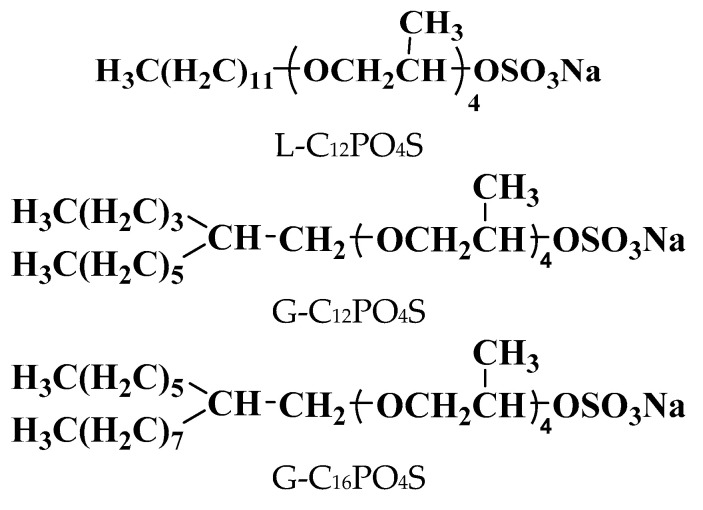
The structures and abbreviations of the extended anionic surfactants.

The linear sodium dodecyl(polyoxyisopropene)_4_ sulfate (L-C_12_PO_4_S), branched sodium dodecyl(polyoxyisopropene)_4_ sulfate (G-C_12_PO_4_S), and branched sodium hexadecyl(polyoxyisopropene)_4_ sulfate (G-C_16_PO_4_S) were provided by the Sasol corporation (South Africa). The molecular structures of the three surfactants are depicted in Scheme 1. Ultrapure water with 18.2 MΩ·cm resistivity was used for the experiments.

### 2.2. Surface Tension Measurements

The surface tension values of the surfactant solutions were detected using an interfacial tension meter (DCAT21, Dataphysics Company, Germany) at 30 °C via the Wilhelmy plate technique. [32] Each concentration was measured three times. The measurement error of the surface tension is lower than 0.5 mN/m.

### 2.3. Contact Angle Measurements

Before the contact angle test, the PMMA surface was cleaned according to the literature [26]. The contact angles on PMMA surface were conducted by the LAUDA Scientific GmbH machine (Lauda-Königshofen, Germany) via the sessile drop method at 30 °C. Each concentration was measured at least five times with 2 μL droplets.

## 3. Results and Discussion

### 3.1. Surface Activity Parameters of the Extended Surfactants

**Figure 1:**
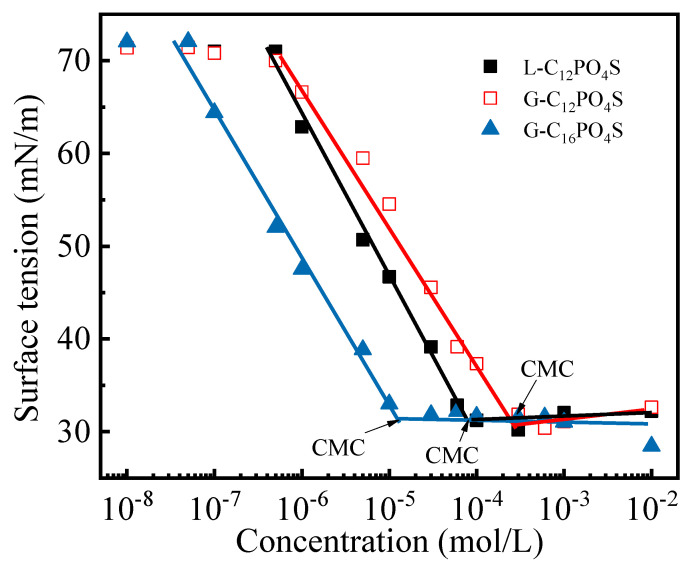
Surface tension (*γ*_LV_) versus the concentration for the extended surfactants.

The surface tensions of the three extended surfactants were measured at different concentrations, and the isotherms of equilibrium surface tensions as a function of concentration are plotted in Figure 1. The CMC value was obtained from the turning point in the curve. CMC is the most critical turning concentration of the surfactant solution, which implies the maximum monomer concentration in the solution. Besides, the surface tension of the surfactant solution at the turning point is represented as *γ*_CMC_. As shown in the surface tension curves (Figure 1), the CMC values of L-C_12_PO_4_S, G-C_12_PO_4_S, and G-C_16_PO_4_S are 9.9 × 10^−5^, 3.0 × 10^−4^, and 1.5 × 10^−5^ mol L^−1^, respectively. It can be seen that the CMC value of G-C_12_PO_4_S is higher than that of L-C_12_PO_4_S. This phenomenon can be attributed to the fact that branched chains will enhance the water-solubility of surfactants. Besides, branched G-C_12_PO_4_S has larger steric hindrance for forming micelles, which will increase the CMC value as well. As for the branched surfactants (G-C_12_PO_4_S and G-C_16_PO_4_S), G-C_16_PO_4_S with enhanced oleophilicity possesses smaller CMC.

The saturated adsorption amount (*Γ*_max_) and saturated adsorption area (*A*_min1_) can be calculated by Gibbs equations as follows

(1) $$\Gamma_{\max}=\;-\;(\frac{1}{2.303\mathrm{nRT}})(\frac{d\gamma }{d\;\log\;C})$$

(2) $$A_{\min}=\frac{10^{14}}{N_{A}\Gamma_{\max}}$$

The values of *Γ*_max1_ and *A*_min1_ are calculated and listed in Table 1. Overall, there are few differences in the surface tension at the CMC (*γ*_CMC_) for the three surfactants. However, the *Γ*_max1_ of the branched G-C_12_PO_4_S is relatively low, resulting in a slightly higher *γ*_CMC_. As the alkyl chain gets longer, the hydrophobicity enhances while the steric hindrance rises. As a result, the *A*_min1_ of the branched surfactants (G-C_12_PO_4_S and G-C_16_PO_4_S) are both approximately 1.33 nm^2^.

Moreover, some reported [29,33] parameters of extended anionic surfactants with similar structures are also listed in Table 1. From the CMC values of the extended surfactants sodium dodecyl polypropylene oxide sulfate (C_12_P_n_S) and sodium nonylphenoxy polypropyleneoxide sulfates (PP_n_S), it can be seen that their CMC values decrease with the increase in the PO units. This can be attributed to the increasing lipophilicity as the PO chain gets longer. By comparing, it is observable that the surface activity parameters of L-C_12_PO_4_S, G-C_12_PO_4_S, and G-C_16_PO_4_S are reasonable.

### 3.2. Contact Angles of the Extended Surfactants at PMMA Surface

**Figure 2:**
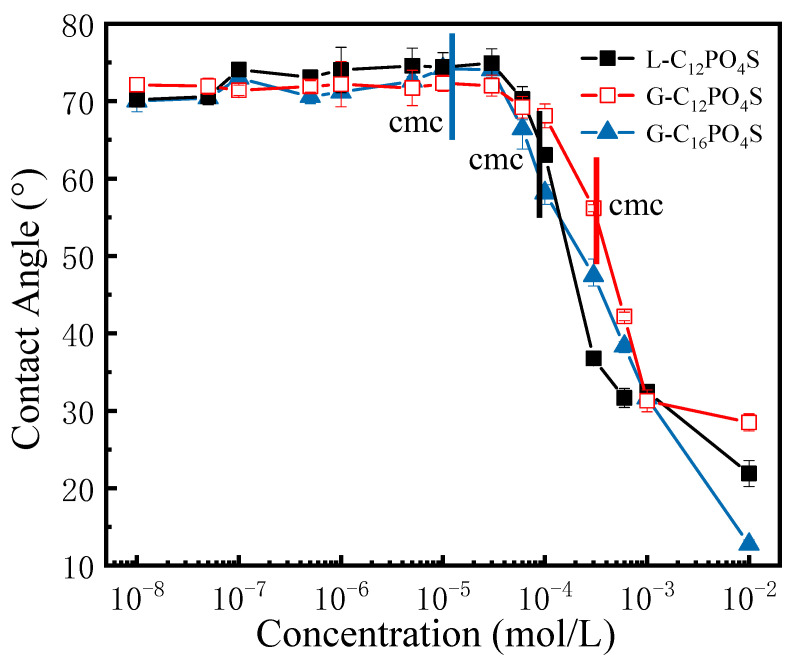
Effect of concentration on contact angles (*θ*) of the extended surfactant solutions on polymethylmethacrylate (PMMA) surface.

Figure 2 shows the contact angles (*θ*) of the three extended surfactant solutions on PMMA surface. It can be seen from Figure 2 that the contact angles of the surfactants vary little over a wide range of concentrations (1 × 10^−8^~3 × 10^−5^ mol L^−1^) with a value of 72°. When the concentration of the surfactant solutions approaches 6 × 10^−5^ mol L^−1^, the contact angles begin to decrease significantly. At high concentration of 1 × 10^−2^ mol L^−1^, the contact angle of linear L-C_12_PO_4_S (21.9°) is smaller than that of branched G-C_12_PO_4_S (28.5°), but higher than that of G-C_16_PO_4_S (12.8°). It also must be pointed out that the *θ* values all decrease obviously after CMC for extended surfactants.

Significantly, the variation in contact angles for these extended anionic surfactants at PMMA surface is quite different from those of conventional surfactants. According to the literature, the contact angle of the cationic surfactant CTAB solution at PMMA surface will decrease dramatically to approximately 51.0° at the CMC, and then change slowly with the increase in bulk concentration [34,35]. The contact angles of anionic surfactant SDS [35] and nonionic surfactant TX-100 [36] decrease to 47.2° and 47.4°, respectively, at the CMC, whereas our previous studies found that the contact angles of special structural surfactants with branched hydrophobic chains or EO groups change noticeably after CMC on the PMMA [24,26,28], quartz [25,32,37], and PTFE [13,38,39] surfaces. For the extended surfactants in this study, the contact angle values also change after CMC, which means that the adsorption of extended surfactant molecules at the solid–liquid interface continues when the adsorption at the liquid surface reaches saturation.

Comparing Figure 1 and Figure 2, surface tensions decrease significantly when contact angles remain constant at a low concentration range. Therefore, the contact angle is not enough to illustrate the adsorption behaviors of surfactants on solid surfaces. The relevant mechanism will be discussed in detail later.

### 3.3. Adhesional Tension of the Extended Surfactants at PMMA–Liquid Interface

The surfactants’ adhesional tension that reveals their adhesion capacity on the solid–liquid interface is defined as the difference between the interfacial free energy of solid–air (*γ*_SV_) and the interfacial free energy of solid–liquid (*γ*_SL_). Based on Young’s equation, the contact angle is related to *γ*_SV_, *γ*_SL_, and *γ*_LV_ (interfacial free energy of solid–liquid, surface tension). Therefore, the value of the adhesional tension is *γ*_LV_cos *θ* (Equation (3)).

(3) $$\gamma_{\mathrm{SV}}-\gamma_{\mathrm{SL}}=\gamma_{\mathrm{LV}}\cos\;\theta$$

To quantify the surfactants’ adsorption amounts at the interfaces, Young’s equation is combined with the Gibbs adsorption equation, and the obtained equation is as follows

(4) $$\frac{d\left(\gamma_{\mathrm{LV}}\;\cos\theta \right)}{d\gamma_{\mathrm{LV}}}=\frac{\Gamma_{\mathrm{SV}}-\Gamma_{\mathrm{SL}}}{\Gamma_{\mathrm{LV}}}$$

where *Γ*_SV_, *Γ*_SL_, and *Γ*_LV_ represent the surfactants’ adsorption amounts at solid–air, solid–liquid, and air–liquid interfaces, respectively. Assuming that *Γ*_SV_ ≈ 0, the value of *Γ*_SL_/*Γ*_LV_ can be obtained from the slope of the *γ*_LV_cos *θ-γ*_LV_ curve.

**Figure 3:**
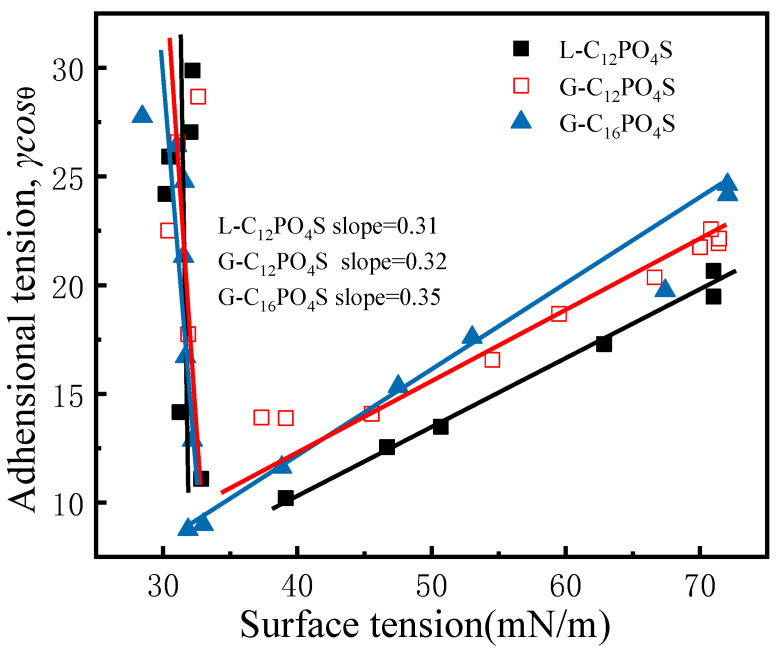
Dependence between the adhesional tension and surface tension of the extended surfactants.

Figure 3 displays the dependence between the adhesional tension and surface tension of the three extended surfactants. Before CMC, the adhesional tension decreases with the increase in bulk concentration. When the bulk concentration exceeds CMC, the surface tension keeps a constant value, while the adhesional tension continues to increase. As a result, the curves exhibit a vertical upward trend. It is noticeable that the surface tension and adhesional tension of the surfactant solutions exhibit a good linear relationship before CMC, and the linear correlation coefficient (R^2^) for L-C_12_PO_4_S, G-C_12_PO_4_S, and G-C_16_PO_4_S is respectively 0.996, 0.990, and 0.973. Interestingly, the slopes of the three extended surfactants are all about 0.3, which means the extended surfactants that adsorb at the air–liquid interface are all approximately three times higher than those at the PMMA–liquid interface.

For comparison, the slopes of the adhesional tension curves of some conventional and special structural surfactants are summarized in Table 2. Based on the value of *Γ*_SL_/*Γ*_LV_ and the surfactants’ saturated adsorption area (*A*_min1_) at the air–liquid interface, the saturated adsorption area (*A*_min2_) of the surfactant molecules on the PMMA surface before CMC is calculated and also listed in Table 2.

In general, the slopes of conventional surfactants such as nonionic surfactant TX-100, [21] anionic surfactant SDDS, [23] and cationic surfactants CTAB, [34] CPyB, [34] C_12_(EDMAB) [19], and BDDAB^19^ are all negative, demonstrating that they absorb at the PMMA-water interface via hydrophobic interactions. Meanwhile, the conventional surfactants exhibit slopes of about −0.3, which indicates that they are likely to tile at the PMMA–liquid interface with about 1/3 of the adsorption amounts at the air–liquid interface. Therefore, the adsorption amounts of both the conventional surfactants (slopes ≈ −0.3) and the extended surfactants (slopes ≈ 0.3) in this work at the air–liquid interface are approximately three times higher than those at the solid–liquid interface. However, the adsorption behavior between the conventional surfactants and the extended surfactants are quite different. However, it must be pointed out that the slope values of the extended surfactants in this work are all positive at a low concentration, which means that surfactant molecules adsorb on the PMMA surface by ionic heads with the hydrophobic tail towards the water.

Comparing the structures of the surfactants, it is worth noting that these specific surfactants with positive slopes have more than one hydrophilic group. As for the three extended surfactants in this work, they all have a PO chain and -SO_4_^−^ group. Besides, the C_16_PC^27^ and C_16_GPC^28^ surfactants have the -OH and -N(CH_3_)_3_^+^ groups. The C_16_PB^27^ and C_16_GPB^28^ molecules have the -OH, -N(CH_3_)_3_^+^ and -COO^−^ groups. The C_16_(EO)_3_PC^27^ and C_16_G(EO)_3_PC^28^ molecules have the -OH, -N(CH_3_)_3_^+^ and EO groups. The C_16_(EO)_3_PB^27^ and C_16_G(EO)_3_PB^28^ surfactants have the -OH, -N(CH_3_)_3_^+^, EO and -COO^−^ groups. Furthermore, the C_3_^26^ and C_6_^26^ surfactants all have two polar xylyl and two -N(CH_3_)_3_^+^ groups. The 18C^40^ molecule has -N(CH_3_)_3_^+^ and -COO^‒^ groups, while the 18S^40^ molecule has -N(CH_3_)_3_^+^, -OH and -SO_3_^−^ groups. Moreover, the BCB^24^ molecule has the polar xylyl, -N(CH_3_)_3_^+^ and -COO^-^ groups, while BSB^24^ molecule has the polar xylyl, -N(CH_3_)_3_^+^, -OH and -SO_3_^−^ groups.

Multi-hydrophilic groups can undoubtedly increase the polar interactions with the PMMA surface. Therefore, these surfactants can adsorb on the PMMA surface by polar interactions that more easily rely on their special structures (hydroxyl, EO, or PO groups). Since the hydroxyl group can form hydrogen bonds, EO group has a good hydrophilic effect, and PO group owns a partly hydrophilic and partly lipophilic nature; these special structural surfactants can easily interact with PMMA surfaces.

The size of hydrophilic part controls the positive value of the slope. As for C_16_(EO)_3_PC and C_16_(EO)_3_PB in Table 2, their -(OCH_2_CH_2_)_n_ groups increase the steric hindrance and reduce their adsorption capacity [27]. This is the reason why their slopes are only 0.033~0.063. Comparing the two linear betaines C_16_PB^27^ and 18C^40^, the slope of C_16_PB is only 0.136 due to its ether hydroxypropyl. Among them, the PO group has a stronger interaction with the PMMA surface due to its partially hydrophilic and partially lipophilic nature [31], so the extended surfactants in this work have the maximum slope values compared with other surfactants with special structures.

### 3.4. Interfacial Tension of the PMMA–Liquid Interface

**Figure 4:**
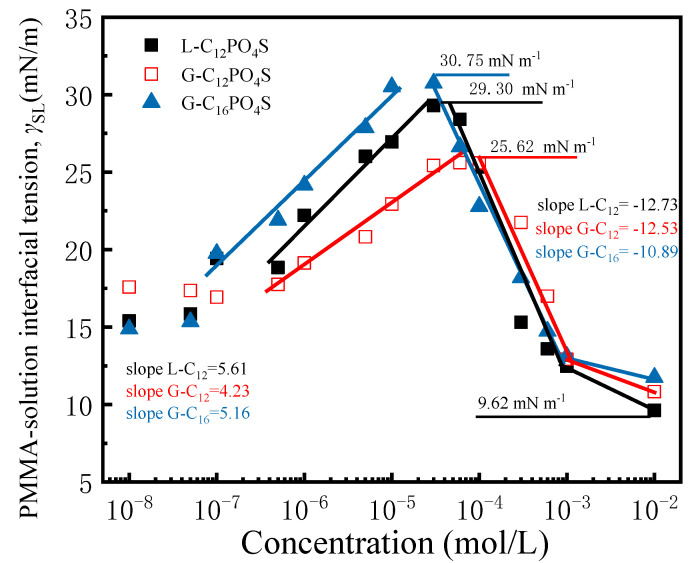
The PMMA–solution interfacial tension as a function of surfactant concentration.

The variation of solid–liquid interfacial tension reveals the adsorption behavior of extended surfactant molecules on a solid surface. The surface free energy of PMMA is about 39.5 mN m^−1^, [27] and the interfacial tension at PMMA-liquid interface (*γ*_SL_) can be calculated by using Young’s equation, and the obtained results are plotted in Figure 4. The saturated adsorption area before CMC (*A*_min3_) and after CMC (*A*_min4_) are calculated by Gibbs equation and listed in Table 3.

As seen in Figure 4, the adsorption of the three extended surfactants on PMMA surface has two stages. At a low concentration, *γ*_SL_ increases gradually with the increase in the bulk concentration. At a high concentration, *γ*_SL_ decreases sharply as the bulk concentration increases. A linear relationship exists between the concentration and *γ*_SL_ in the two stages. There is a maximal *γ*_SL_ in the PMMA–solution interfacial tension curve for each surfactant, and the values of *γ*_SL_ vary with the structure of extended surfactants.

In the first stage, the extended surfactants interact with PMMA surface by polar groups, so *γ*_SL_ gradually increases. As a consequence, the hydrophobic tails that point to water strengthen the hydrophobic modification of the PMMA surface. Because of the electrostatic repulsion among the surfactant molecules, the adsorption amount on the solid surface at this stage is low (Table 3). Though the ionic group SO_4_^−^ is followed by the PO group, the steric hindrance of the PO group does not work due to the small adsorption amount of the surfactants. On account of the higher steric hindrance, the branched G-C_12_PO_4_S has a smaller *Γ*_max_ (3.6 × 10^−11^ mol cm^−2^) and a larger *A*_min_ (4.56 nm^2^) than L-C_12_PO_4_S. Besides, the longer alkyl chain results in a stronger hydrophobic interaction. Accordingly, G-C_16_PO_4_S has a larger adsorption amount and smaller adsorption area than G-C_12_PO_4_S. Furthermore, the values of *A*_min3_ calculated by the Gibbs equation are consistent with the theoretical *A*_min2_ obtained by adhesional tension, which implies the reliability of the results.

In the second stage, the hydrophobic tails of the surfactants interact with the PMMA surface, while the hydrophilic heads point to the solution. Consequently, the solid surface is modified to be hydrophilic, and *γ*_SL_ decreases significantly when the bulk concentration increases. Combined with the previous research, the change trends of *γ*_SL_ have three cases as the surfactant concentrations increase: (1) *γ*_SL_ rises firstly and then keeps a stable value; (2) *γ*_SL_ rises firstly and then declines, and the slopes of the two stages are almost equal; (3) *γ*_SL_ rises firstly and then declines, and the slope of the second stage is 2–3 times higher than the first stage. Zhang et al. [24] studied the wettability of benzyl-substituted alkyl sulfobetaine (BSB) on PMMA surface. It is found that the *γ*_SL_ values increases as the bulk concentration increases and then *γ*_SL_ reaches a plateau value around CMC, which indicates that BSB molecules adsorb on PMMA surface only by polar interaction. Therefore, the hydrophobic adsorption stage does not occur. Hu et al. [40] investigated the adsorption of alkyl carboxylbetaine (18C) and alkyl sulfobetaine (18S) on the PMMA surface. They found that the *γ*_SL_ rises firstly and then declines, and the slope of the decreasing stage is almost the same as the increasing stage. The saturated adsorption areas of surfactants (18C, 18S) on the PMMA–liquid interface before CMC (*A*_min3_) are, respectively, 2.78 and 3.06 nm^2^, which is very close to the *A*_min4_ after CMC (2.99 nm^2^ and 4.57 nm^2^). This implies that the hydrophobic chains of the surfactants are towards the water at the beginning, but the surfactants may form the bilayer adsorption film with the hydrophilic heads towards the water as the bulk concentration increases. Lv et al. [26] found a similar bilayer adsorption phenomenon on the wettability of xylyl-substituted biquaternary ammonium salt Gemini surfactants (C3, C6) on PMMA surface. In this work, it is obvious that the slopes of *γ*_SL_ after CMC are much larger than the slopes at a low concentration, which demonstrates that the adsorption amount of the surfactants on the PMMA surface remarkably increase. Therefore, the adsorbed surfactants are more likely to form aggregations on the solid surface than bilayer adsorption.

At the turning point of adsorption behavior, the maximum value of *γ*_SL_ represents the maximum hydrophobic modification capability of the extended surfactants. It is visually observable that branched G-C_16_PO_4_S whose *γ*_SL_ reaches 30.75 mN∙m^−1^ possesses the strongest hydrophobic modification ability. Furthermore, the minimum value of *γ*_SL_ at high concentration can manifest the maximum hydrophilic modification ability of the surfactants. It can be seen that *γ*_SL_ of linear L-C_12_PO_4_S reaches 9.62 mN∙m^−1^, suggesting that L-C_12_PO_4_S has the strongest hydrophilic modification capability.

The maximum hydrophobic and hydrophilic modification abilities of the special structural surfactants in the literature are summarized in Table 4. In order to avoid the influence of the initial values of *γ*_SL_ for different PMMA sheets in the literature, the maximum hydrophobic modification capacity (Δ*γ*_SL_) is represented by the difference between the maximum *γ*_SL_ and the initial *γ*_SL_ for pure water, whereas the maximum hydrophilic modification capacity corresponds to the minimum *γ*_SL_ at high concentrations. It is observable that L-C_12_PO_4_S, G-C_16_PO_4_S, BCB, and BSB possess strong hydrophobic modification ability with Δ*γ*_SL_ of more than 10 mN∙m^−1^. Comparing their structures, the strong hydrophobic modification ability can be attributed to the strong polar interactions and the small steric hindrance. On one hand, the stronger the polar interactions, the more surfactants will adsorb at the PMMA surface. On the other hand, less steric hindrance leads to relatively higher adsorption amounts, thus manifesting strong hydrophobic modification ability. The branched C_16_GPB, C_16_GPC, and C_16_G(EO)_3_PC have a higher hydrophilic modification ability on the PMMA surface compared to the linear surfactants with similar structures. However, the branched structure increases the difficulty of forming aggregations on the PMMA surface, thus the hydrophilic modification ability of C_16_G(EO)_3_PB is lower than that of the linear C_16_(EO)_3_PB.

### 3.5. Adhesion Work of the Extended Surfactants on PMMA Surface

The work of adhesion (*W*_A_) of the surfactant solutions on the solid surface, which can represent the work required to separate a unit area of liquid from the solid surface, can be computed by Equation (5). Moreover, combined with Young’s equation, *W*_A_ can be obtained as shown in Equation (6).

(5) $$W_{A}=\gamma_{\mathrm{SV}}+\gamma_{\mathrm{LV}}-\gamma_{\mathrm{SL}}$$

(6) $$W_{A}=\gamma_{\mathrm{LV}}\left(\cos\theta +1\right)$$

According to the equation, the adhesion work is the sum of adhesional tension (*γ*_LV_cos *θ*) and surface tension (*γ*_LV_).

**Figure 5:**
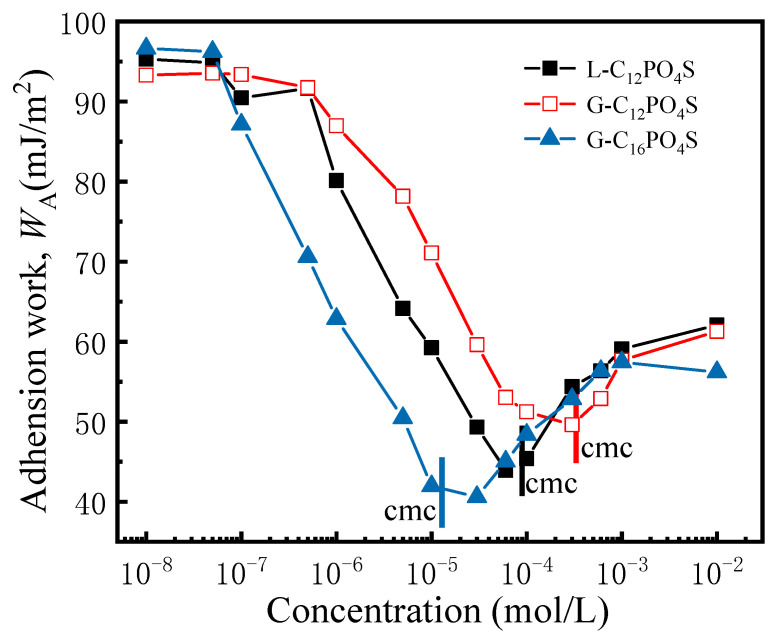
Effect of concentration on the adhesion work of the extended surfactants at the PMMA surface.

The work of adhesion (*W*_A_) for the extended surfactants at the PMMA surface has been calculated and plotted in Figure 5. We can see from Figure 5 that *W*_A_ of the surfactant solutions is almost constant at low concentrations because the *γ*_LV_ and *γ*_SL_ change little. As the concentration increases, *W*_A_ decreases, which is caused by the decrease in both *γ*_LV_ and adhesional tension. When the concentration exceeds the CMC value, the adsorption of surfactants at the air–liquid interface is saturated and the *γ*_LV_ is constant. However, the surfactants continue to adsorb at the PMMA–liquid interface and, accordingly, the *γ*_SL_ decreases, which leads to an increasing trend of *W*_A_.

### 3.6. Adsorption Mechanism of the Extended Surfactants on PMMA Surface

**Figure 6:**
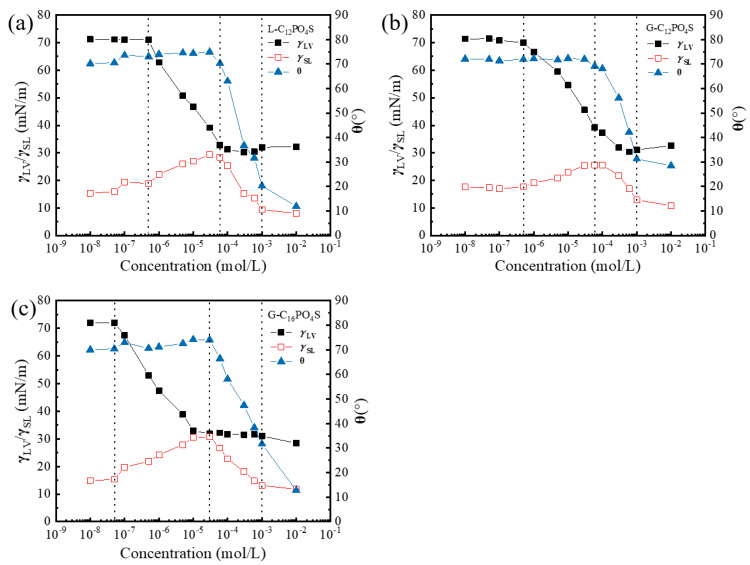
Concentration dependence of the adhesion data of extended surfactants L-C_12_PO_4_S (**a**), G-C_12_PO_4_S (**b**), and G-C_16_PO_4_S (**c**) on PMMA surface.

**Figure 7:**
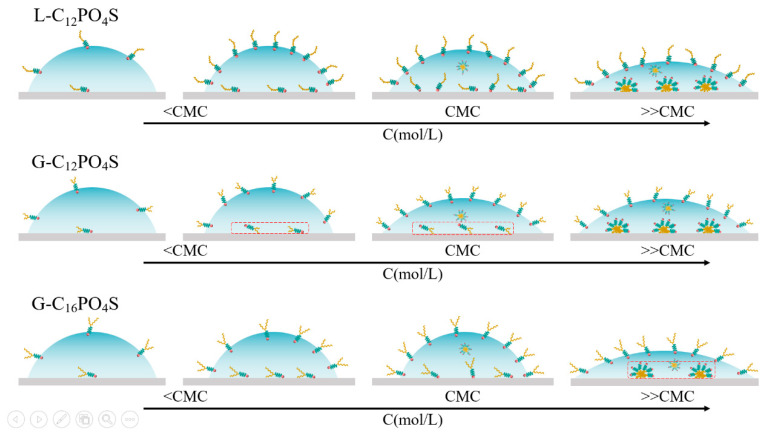
The possible adsorption behaviors of the surfactants on the PMMA surface.

The mechanisms responsible for the adsorption behaviors and wettability of the extended anionic surfactants were detected by analyzing their structural dependence results in Figure 6, and the corresponding possible adsorption behaviors are schematically plotted in Figure 7.

Observing the curves in Figure 6, it can be found that the adsorption behaviors of the three extended surfactants on the PMMA surface can be divided into four stages. We take linear L-C_12_PO_4_S as an example to discuss the four stages of the surfactants’ adsorption behavior.

In the first stage (1 × 10^−8^~5 × 10^−7^ mol L^−1^), the adsorption of surfactant molecules at both the solid–liquid interface and the air–liquid interface can be ignored, and the contact angles remain about 72° and the other adhesion data keep constant.

In the second stage (5 × 10^−7^~6 × 10^−5^ mol L^−1^), the adsorption amounts at both the air–liquid and solid–liquid interface increase as the bulk concentration increases, which results in a decreasing *γ*_LV_ and an increasing *γ*_SL_. However, the contributions of *γ*_LV_ and *γ*_SL_ are offset, resulting in little change in the contact angles. At the same time, surfactants rely on ionic heads to adsorb at the solid–liquid interface, and the hydrophobic chains point toward the solution. The contact angles exhibit a slightly increasing tendency on account of the hydrophobized PMMA surface.

In the third stage (6 × 10^−5^~1 × 10^−3^ mol L^−1^), the surfactants’ adsorption amounts at the air–liquid interface tend to be saturated and the *γ*_LV_ tends to reach a plateau value when approaching the CMC value. However, the *γ*_SL_ suddenly decreases, which triggers a sharp decline in the contact angles. The adsorption behavior starts to transform, and surfactant molecules are more inclined to adsorb on the PMMA surface through hydrophobic interactions. Since the adsorption amounts of surfactant molecules increase significantly at this stage, it is presumed that the adsorbed surfactants are forming aggregations at the solid–liquid interface, as we discussed above.

At the last stage (1 × 10^−3^~1 × 10^−2^ mol L^−1^), the adsorbed surfactant molecules at the solid–liquid interface also tend to be saturate; thus, the contact angles display a stable tendency.

Among the three surfactants, the *γ*_SL_ of L-C_12_PO_4_S and G-C_16_PO_4_S starts to decrease when the surfactant concentration exceeds CMC. Similarly, the contact angles of L-C_12_PO_4_S and G-C_16_PO_4_S start to decrease after CMC. Interestingly, the *γ*_SL_ of G-C_12_PO_4_S starts to decrease before CMC, indicating that its adsorption behavior changes before CMC. The branched chain of the G-C_12_PO_4_S molecules enhanced the hydrophobic effect, thus intensifying the adsorption of hydrophobic groups on the PMMA surface. On the other hand, a branched chain improves the water-solubility of G-C_12_PO_4_S and CMC value increases as a result. Therefore, the G-C_12_PO_4_S molecules begin adsorbing on PMMA surface before the saturated adsorption at the air–liquid interface. Meanwhile, the G-C_12_PO_4_S molecules start to form aggregations like semi-micelles at the PMMA–liquid interface. As the branched chains get longer, however, the steric hindrance at PMMA surface increases. It becomes difficult to form aggregations at a solid surface, so the *γ*_SL_ of G-C_16_PO_4_S changes after CMC. Moreover, G-C_16_PO_4_S shows the highest *γ*_SL_ value at high concentrations, which can also be attributed to the steric hindrance to forming aggregations.

For the extended surfactants, the branched G-C_16_PO_4_S molecules with two long alkyl chains exhibit the strongest hydrophobic modification capacity. Due to its having the least steric hindrance and the highest adsorption amount, the linear L-C_12_PO_4_S is more likely to form aggregations at the PMMA–liquid interface. Therefore, L-C_12_PO_4_S manifests a stronger hydrophilic modification ability than branched G-C_12_PO_4_S and G-C_16_PO_4_S surfactants.

## 4. Conclusions

In this work, the adsorption mechanism and wetting properties of branched extended anionic surfactants (L-C_12_PO_4_S, G-C_12_PO_4_S, G-C_16_PO_4_S) on the PMMA surface were investigated. Based on the structural dependence of extended anionic surfactants on the wettability of PMMA surface, the following conclusions are obtained:

(1) Before CMC, the contact angles change little as the surfactants’ concentration increases because the decrement of γ_LV_ exactly counterbalances the increment of γ_SL_. The contact angles decrease significantly after CMC, which attributes to the rapidly decreasing γ_SL_;

(2) Before CMC, the adhesional tension decreases with the increase in bulk concentration, and a linear relationship exists between the adhesional tension and surface tension. After CMC, the adhesional tension exhibits a vertical upward trend in the *γ*_LV_cos*θ*-*γ*_LV_ curve. At low concentrations, the special property of PO groups allows the extended surfactants to adsorb on the PMMA surface by polar interactions; thus, the solid surface is modified to be hydrophobic. Besides this, the surfactants’ adsorption amounts at the air–liquid interface are all approximately three times higher than those at the PMMA–liquid interface before CMC;

(3) There are two stages for the three extended surfactants in PMMA–solution interfacial tension. At low concentrations, γ_SL_ increases gradually with the increase in the bulk concentration. At high concentrations, γ_SL_ decreases significantly as the bulk concentration increases. In this stage, the surfactants are prone to forming aggregations and their hydrophobic tails will interact with the PMMA surface with their polar heads pointing to the water. Accordingly, the PMMA surface is modified to be hydrophilic;

(4) In the three extended surfactants, linear L-C_12_PO_4_S with the least steric hindrance shows the strongest hydrophilic modification ability. On the contrary, the branched G-C_16_PO_4_S molecules with two long alkyl chains exhibit the strongest hydrophobic modification capacity.

## Figures and Tables

**Table 1:** The critical micelle concentration (CMC), *γ*_CMC_, and saturated adsorption amount (*Γ*_max1_) and area (*A*_min1_) of the surfactants.

Abrr.	CMC/(10^−5^ mol L^−1^)	*γ*_CMC_/(mN m^−1^)	10^10^*Γ*_max1_/(mol cm^−2^)	*A*_min1_/(nm^2^)
L-C_12_PO_4_S	9.9	30.1	1.47	1.13
G-C_12_PO_4_S	30.0	31.4	1.25	1.33
G-C_16_PO_4_S	1.5	30.7	1.24	1.34
C_12_P_4_S^33^	42.0	40.0	1.00	1.66
C_12_P_8_S^33^	7.8	35.5	1.08	1.54
C_12_P_12_S^33^	4.3	33.7	1.01	1.64
PP_3_S^29^	5.9	34.4	1.66	1.00
PP_6_S^29^	3.0	34.1	1.29	1.29
PP_9_S^29^	1.6	33.7	1.17	1.41
PP_12_S^29^	1.2	36.9	0.97	1.71

**Table 2:** The slopes (*Γ*_SL_/*Γ*_LV_) and the theoretical adsorption area (*A*_min2_) of the conventional surfactants and several surfactants with specific structures before CMC.

Surfactants	Slopes	*A*_min2_/(nm^2^)
L-C_12_PO_4_S	0.31	3.65
G-C_12_PO_4_S	0.32	4.16
G-C_16_PO_4_S	0.35	3.83
CTAB^34^	−0.34	-
CPyB^34^	−0.34	-
C_12_(EDMAB)^19^	−0.30	-
BDDAB^19^	−0.31	-
C_16_PC^27^	0.045	-
C_16_(EO)_3_PC^27^	0.033	-
C_16_PB^27^	0.136	-
C_16_(EO)_3_PB^27^	0.063	-
C_16_GPC^28^	0.11	7.36
C_16_G(EO)_3_PC^28^	0.11	6.36
C_16_GPB^28^	0.11	6.00
C_16_G(EO)_3_PB^28^	0.11	6.18
C_3_^26^	0.13	8.69
C_6_^26^	0.13	9.62
18C^40^	0.26	2.84
18S^40^	0.28	3.01
BCB^24^	0.30	2.00
BSB^24^	0.34	2.05

**Table 3:** The surface activity parameters of the extended surfactants at PMMA.

Abbr.	10^10^*Γ*_max3_/(mol/cm^2^) (<CMC)	*A*_min3_/(nm^2^) (<CMC)	10^10^*Γ*_max4_/(mol/cm^2^) (>CMC)	*A*_min4_/(nm^2^) (>CMC)
L-C_12_PO_4_S	0.48	3.44	1.10	1.51
G-C_12_PO_4_S	0.36	4.56	1.08	1.54
G-C_16_PO_4_S	0.44	3.74	0.94	1.77

**Table 4:** The maximum hydrophobic modification and the maximum hydrophilic modification of the special surfactants on PMMA surface in the literature.

Surfactants	Δ*γ*_SL_	The Minimum *γ*_SL_
L-C_12_PO_4_S	13.91	9.62
G-C_12_PO_4_S	8.69	10.83
G-C_16_PO_4_S	15.87	11.75
C_16_PC^27^	1.1	21.75
C_16_(EO)_3_PC^27^	4.25	26.8
C_16_PB^27^	5.4	27.2
C_16_(EO)_3_PB^27^	4.1	9.75
C_16_GPC^28^	2.7	18.8
C_16_G(EO)_3_PC^28^	4.4	18
C_16_GPB^28^	5	18
C_16_G(EO)_3_PB^28^	4.9	17.5
C_3_^26^	2.8	29.8
C_6_^26^	3	28.1
18C^40^	9	21
18S^40^	9.2	24
BCB^24^	11.8	22.5
BSB^24^	13	27.8

## Data Availability

Not applicable.

## Sample Availability

Samples of the L-C_12_PO_4_S, G-C_12_PO_4_S, and G-C_16_PO_4_S are available from the authors.
